# A Combination of Podophyllotoxin and Rutin Attenuates Radiation Induced Gastrointestinal Injury by Negatively Regulating NF-κB/p53 Signaling in Lethally Irradiated Mice

**DOI:** 10.1371/journal.pone.0168525

**Published:** 2016-12-30

**Authors:** Bhargab Kalita, Rajiv Ranjan, Abhinav Singh, M. H. Yashavarddhan, Sania Bajaj, Manju Lata Gupta

**Affiliations:** Division of Radioprotective Drug Development and Research, Institute of Nuclear Medicine and Allied Sciences, Brig.S.K Mazumdar Marg, Delhi, INDIA; National Cheng Kung University, TAIWAN

## Abstract

Development of an effective radio protector to minimise radiation-inflicted damages have largely failed owing to inherent toxicity of most of the agents examined so far. This study is centred towards delivering protection to lethally irradiated mice by pre-administration of a safe formulation G-003M (combination of podophyllotoxin and rutin) majorly through regulation of inflammatory and cell death pathways in mice. Single intramuscular dose of G-003M injected 60 min prior to 9 Gy exposure rescued 89% of whole body lethally irradiated C57BL/6J mice. Studies have revealed reduction in radiation induced reactive oxygen species (ROS), nitric oxide (NO) generation, prostaglandin E2 (PGE2) levels and intestinal apoptosis in G-003M pre-treated mice intestine. Restricted nuclear translocation of redox-sensitive Nuclear factor-κB (NF-κB) and subsequent downregulation of cyclo-oxygenase 2 (COX-2), inducible nitric oxide synthase (iNOS; EC 1.14.13.39) and tumor necrosis factor (TNF-α) levels demonstrated the anti-inflammatory effect that G-003M exerts. Support to early hematopoietic recovery was exhibited through G-003M mediated induction of granulocyte colony stimulating factor (G-CSF) and interleukin (IL-6) levels in lethally irradiated mice. Considerable attenuation in radiation induced morphological damage to the intestinal villi, crypts and mucosal layers was observed in G-003M pre-treated mice. Additionally, our formulation did not reduce the sensitivity of tumor tissue to radiation. Altogether, these results suggest that G-003M ameliorates the deleterious effects of radiation exposure by minimising ROS and NO generation and effectively regulating inflammatory and cell death pathways. Mechanism of protection elucidated in the current study demonstrates that G-003M can be used as a safe and effective radio protective agent in radiotherapy for human application.

## Introduction

Ionizing radiation damages cellular bio molecules either by generating free radicals (indirect effect) or directly depositing energy into cellular entities. Degree of damage is known to be directly proportional to the cell proliferation rate. Hematopoietic system and the gastrointestinal (GI) tract fall into this category due to a constant phenomenon of cell division and differentiation occurring in these organs. Error free functioning of GI and hematopoietic systems is essentially important to prevent radiation induced mortality. Stem cells, though located at different sites in both the organs, yet functions similarly in proliferation, differentiation and renewal of damaged tissue during severe radiation exposure. Crypt injury deranges the functionality of stem cells housed in crypts, leading to villi shortening, nutrient imbalance and damaged intestinal epithelium. These factors collectively disturb the intestinal immune homeostasis resulting in intestinal inflammation [1, 2]. GI damage is among one of the prominent causes for radiation induced fatality. In contrast to hematopoietic (HP) injuries which can be rescued by cytokines administration/bone marrow transplantation, there is no approved therapeutic or preventive measures for GI damage. Hence, protection to GI is a valuable support for medical management not only in case of accidental radiation exposures but also during radiotherapy/chemotherapy protocols [3].

Inflammatory response in GI attributed by radiation induced generation of reactive oxygen/nitrate species which subsequently activates a cascade of signaling pathways through redox-sensitive transcription factors, proinflammatory cytokines resulting into induction of cell death/apoptosis [4, 5, 6]. Separate studies conducted in GI epithelial cell lines have reported concominant increase in Prostaglandin E2 levels following γ -ray irradiationthroughupregulation of Cyclooxygenase (COX-2) expression [7]. Association of inducible Nitric Oxide Synthase (iNOS) with nitric oxide (NO) production in gut epithelium and compromising its function has also been well reported [8]. These suggest an interactive role of NF-κB, COX-2 and iNOS in regulating inflammatory response in GI during radiation exposure. Radiation is also widely used to arrest tumor growth during anti-cancer therapy [9]. Normal tissue toxicity is the biggest challenge for patients undergoing radiotherapy. Though efforts have been undertaken to develop agents that protect normal tissues without interfering the anti-proliferative and cell-killing effects of radiation in tumor [10], yet the issue is still unresolved.

To alleviate radiation induced intestinal damage, several therapeutic strategies have been tested. However, due to various constraints including undesired toxicity associated with most of the anti-radiation agents, their use have become limited [11]. Owing to the potent anti-oxidant and anti-inflammatory properties, exploration on natural resources has become one of the most promising themes to counter the deleterious effects of radiation [12]. Besides, several herbs evaluated to minimize radiation induced injuries, the whole extract and semi purified fractions derived from *Podophyllum hexandrum* were found to confer significant survival benefits to radiation exposed rodent models [13, 14]. After observing radio protective potential in various solvent extracts, we isolated several active molecules from *Podophyllum hexandrum* rhizomes and chemically characterised them [15]. Their bioactive characteristics against radiation have shown that a combination of three isolated active principles could abrogate radiation induced DNA-damage response in human peripheral blood lymphocytes [16, 17]. Radiation injury caused to mice lungs [18] and hepatic systems [19] were also minimized. In addition, the formulation has also been reported to improve recovery of radiation induced injuries by co-ordinated regulation of oxidative stress signaling pathway [20].

The present study was undertaken to assess the anti-inflammatory property of our radio protective formulation (G-003M) prepared by combining two molecules podophyllotoxin and rutin. The study includes G-003M mediated modulation in ROS and NO generation, NF-κB expression and its translocation to the nuclear region in jejuna tissues of differentially treated mice. Further, the regulation of inflammatory entities such as COX-2, iNOS in jejuna epithelium and serum cytokine levels were also explored. Expression of apoptosis response markers Bax, Bcl2, p53, PUMA and cell death was also evaluated. Treatment dependent changes to the villi, crypts and the mucosal layer was ascertained by conventional histopathology. Additionally, potential of G-003M uses in cancer radiotherapy for differential protection in normal tissues versus tumor in mice model was also envisaged.

## Materials and Methods

### Experimental animals and γ-irradiation

MaleC57BL/6J (6–8 weeks old) maintained at the Institute’s experimental animal facility were used in the study. Mice were housed upto six per cage on a 12 hr light/dark cycle in a well maintained pathogen free and temperature (25±2°C) controlled environment and were provided with certified rodent diet and filtered water *ad libitum*. Mice were placed in rectangular boxes with source to sample distance of 120cm and exposed to 9 Gy total body γ- irradiation (TBI) at a dose rate of 1.08–0.955 Gy/min in a ^60^Co source cobalt teletherapy unit (Cobalt Tele Therapy, Bhabatron II). Corresponding control mice group were sham irradiated. After adaptation period of a week, a total of 24 mice were randomized by weight into four groups. The animals appearing unhealthy or losing weight were excluded from the experiments. The four groups consist of (I) Untreated (mice with no treatment, n = 3–6) (II) G-003M (mice treated with G-003M (6.5 mg/kg body wt) alone i.m., n = 3–6) (III) IR (mice treated with radiation, n = 3–6) (IV) G-003M+IR (mice treated with intramuscular injection of G-003M followed by radiation, n = 3–6). Each experiment was repeated 3 times.

### Ethics statement

The study design strictly adhered to the guidelines approved by Institutional Animal Care and Use Committee (IACUC) of our institute, Institute of Nuclear Medicine and Allied Sciences (INMAS) (INM/IAEC/2013/03, dated 06.06.2013). For the survival study period (0 to 30 days) animals were monitored daily for any clinical signs of distress such as weight loss, hunched posture, diarrhoea, abdominal breathing, inability to stand and reduced activity. When an animal was found with deteriorating health condition using the definitive criteria mentioned above, it was humanely euthanized at an early end-point using100% CO_2_ asphyxiation. All efforts were made to minimize animal suffering by administering anaesthesia (intraperitoneal ketamine and xylazine 7:1 mg/ml for 100 l/mouse) prior to euthanasia. All the animal experimental procedures for tumor development in mice were carried out in strict accordance with the recommendations in the Guide for the Care and Use of Laboratory Animals in cancer research of United Kingdom Coordinating Committee on Cancer Research (UKCCCR) and duly approved by the Institutional Animal Care and Use Committee (IACUC). As per the UKCCCR guidelines, animals were sacrificed using cervical dislocation method to avoid tumor burden related discomfort to the animal after tumor size reached a threshold volume.Different group of animals were euthanized by cervical dislocation method at different study end-points. All efforts were made to minimise suffering during animal euthanasia.

### Radioprotective formulation (G-003M)

Radioprotective formulation (G-003M) was prepared by combining purified podophyllotoxin and rutin in 1:2 ratio. Chemical characterization and purity of molecules was confirmed by High Performance Liquid Chromatography (HPLC) and HPTLC. G-003M was injected intramuscularly at its therapeutic concentration of 6.5 mg/kg 60 min before irradiation.

### Survival Assays in mice

For survival assay in mice, four experimental groups with 18 mice each were formed.

Group I: Vehicle (DMSO) + 9 Gy

Group II: G-003M only

Group III: Total Body Irradiation (TBI; 9 Gy)

Group IV: G-003M + 9 Gy TBI

G-003M was injected at its therapeutic concentration through intramuscular route. Group II had only G-003M treatment at its therapeutic concentration. Animals of all the experimental groups were inspected daily for their morbidity and mortality status for a total duration of 30 days. All surviving mice were euthanized humanely at the completion of the observation period.

### Isolation of intestinal epithelial cells (IEC)

Mice were euthanized as per prescribed protocol and the intestinal lumen was flushed with phosphate buffer saline (PBS) at room temperature. The jejuna region of the intestinal lumen was used for isolation of intestinal epithelial cells (IEC). The IEC were isolated as per protocol described in [21, 22]. IEC isolated from the above protocol were used separately for flow cytometry and western blot analysis.

### Intracellular ROS estimation in mice IEC

Level of intracellular ROS production in IEC was estimated using the fluorescent probe 2ʹ, 7ʹ dicholorofluorescein di-acetate (DCF-DA, Sigma, Cat no. 35845-1G). Briefly, after isolation of IEC, cells were pelleted by centrifugation at 1000xg for 10 min, and washed with PBS. Cells were resuspended in 1 ml of PBS, incubated in the dark with the fluorescent probe H_2_DCFDA (final concentration 10 μM) at 37°C for 20 min, and were washed in PBS once. Cells were resuspended in 500 μl of PBS and data acquired by (FACS Calibur 3CB, Becton DickinsonBiosciences, USA) and fluorescent microscope (BX63, Olympus, Japan).

### Isolation of mice peritoneal macrophages

Mice were euthanized prior to the procedure for isolation. After dampening animal fur with 70% EtOH, animal was secured on dissection board. 5 mL sterile PBS was injected into the caudal half of the peritoneal cavity using a 25-gauge needle (bevelled side up). After shaking the entire body for 10 seconds, saline containing resident peritoneal cells was withdrawn by inserting a 19-gauge needle, bevelled side down, into the cranial half of the peritoneal cavity. Briefly, cells were pelleted by centrifugation for 10 min at 300xg and washed twice with RPMI + 10% FCS. Viability was checked by Trypan-blue exclusion (>95% viability). For flow cytometry, cells were resuspended in ice-cold PBS.

### Immunoblot analysis

IEC, peritoneal macrophages and frozen jejunal tissue samples were homogenized in RIPA lysis buffer (Pierce, USA) supplemented with protease inhibitor cocktail (Sigma), and the homogenate centrifuged at 16000xg at 4°C for 20 min. Protein concentration from the supernatant was estimated using the Bradford reagent (Sigma, Cat no. B6916-500ML). Aliquots of total protein (30μg) were denatured and were subjected to SDS-PAGE on 8–12% polyacrylamide gels and were electrophoretically transferred to a Whatman PROTRAN Nitrocellulose Transfer Membrane (Sigma Co., St. Louis, MO). The membranes were blocked for 2 hr with 5% non-fat skimmed milk in PBST (137mM NaCl, 27mM KCl, 10mM Na_2_HPO_4_, 2mM KH_2_PO_4_ and 0.1% Tween20). After blocking nonspecific sites, the membrane was incubated with appropriate primary antibodies mouse anti-NF-κB (1:4000 dilution, Sigma, Cat no. N8523-0.2ML), rabbit anti-IκBα (1:4000 dilution, Millipore, Cat no. 07–1483), goat anti-COX-2 (1:2500 dilution, Santa-Cruz Scientific, Cat no. sc-1747), rabbit anti-iNOS (1:2500 dilution, Santa-Cruz Scientific, Cat no. sc-651), mouse anti-p53 (1:5000 dilution, Millipore, Cat no. CBL404), rabbit anti-PUMA (1:5000 dilution, Millipore, Cat No. AB10418), mouse anti-MDM2 (1:2500 dilution, Millipore, Cat no. 04–1530), mouse anti-p21 (1:4000 dilution, Millipore, Cat no. 05–345), mouse anti-Bax (1:4000 dilution, Sigma), mouse anti-Bcl2 (1:4000 dilution, Sigma), mouse anti β-actin (1:1000 dilution, Santa-Cruz Scientific, Cat no. sc-8432) overnight at 4°C. The membranes were washed in buffer containing PBS plus 0.1% Tween 20 and subsequently incubated with horseradish peroxidise (HRP)-conjugated secondary antibody (1:7000 dilution, Millipore, Cat no. AP307P) for approximately 2 hr at room temperature. Expression signal was detected using enhanced chemiluminescence detection system (Sigma, Cat no. CPS160-1KT). The relative density of the bands in the immunoblot was quantified using BioRad Gel Documentation System (Image Lab Software) and it was normalised to β-actin band intensity.

### Subcellular (nuclear and cytoplasmic) fractionation

Nuclear and cytoplasmic fractions from the jejunum homogenates were prepared using NE-PER Nuclear and Cytoplasmic Extraction Reagents (Pierce, Rockford, IL, Cat no. 78833). Protein concentration was determined by Bradford reagent (Sigma, Cat no. B6916-500ML), using BSA as the standard. The fractions were aliqouted and stored at -80°C. Examination of NF-κB expression in nuclear and cytoplasmic fractions and IκBα expression in the cytoplasmic fractions was done by western blotting as described above by using mouse anti-NF-κB (1:4000 dilution, Sigma, Cat no. N8523-0.2ML), rabbit anti-IκBα (1:4000 dilution, Millipore, Cat no. 07–1483), mouse anti β-actin (1:1000 dilution, Santa-Cruz Scientific, Cat no. sc-8432), anti-Lamin A polyclonal primary antibody (1:4000; Sigma Co., St. Louis, MO), and goat anti-mouse and goat anti-rabbit IgG-HRP secondary antibody (1:7000; Millipore, MA, USA, Cat no. AP308P, AP307P).

### Flow cytometry analysis of iNOS expression in mice peritoneal macrophages

Briefly after isolation of peritoneal macrophages, cells were washed twice with PBS (centrifugation at 1000xg for 10 min) and fixed in 100 μl of 3% paraformaldehyde with an incubation of 30 min in ice. After the fixation step, cells were washed twice with 1 ml PBS followed by washing with 1 ml of 50 mM ammonium chloride. After washing twice with PBS, cells were permeabilized in 200 μl of 0.1% Triton X- 100 in PBS for 20 minutes at 4°C. Blocking step was performed using 10% BSA in PBS for 100 min at RT followed by overnight incubation with rabbit anti-iNOS (1:50 dilution, Santa-Cruz Scientific, Cat no. sc-651). Cells were then washed with PBS and incubated with polyclonal goat anti rabbit FITC conjugated secondary antibody (1:500 dilution, Millipore, Cat no. AP307F)at 4°C. 10,000 cells/sample were analyzed for fluorescence intensity of iNOS by (FACS Calibur 3CB, Becton Dickinson Biosciences, USA).

### Estimation of intracellular nitric oxide (NO) levels in IECs and peritoneal macrophages

Level of intracellular NO production in IECs and mouse peritoneal macrophages was estimated using the fluorescent probe 3-Amino,4-aminomethyl-2′,7′-difluorofluorescein Diacetate (DAF-FM DA, Calbiochem, Millipore, Cat no. 251520). Briefly, after isolation of IEC and mouse peritoneal macrophages, cells were pelleted by centrifugation at 1000xg for 10 min, and washed with PBS. Cells were resuspended in 1 ml of PBS, incubated in the dark with the fluorescent probe DAF-FM DA (final concentration 10 μM) at 37°C for 20–60 min, and were washed in PBS once. Cells were resuspended in 500 μl of PBS and data acquired by flow cytometry (FACS Calibur 3CB, Becton DickinsonBiosciences, USA).

### Estimation of PGE2 levels in intestine

16,16-dimethyl PGE2 (dmPGE2; Sigma, Cat no. D0160) was dissolved in ethanol and diluted into sterile 5% sodium bicarbonate freshly before use. A single dose (0.5 mg/kg) of dmPGE2 was injected intraperitoneally at 1hr before irradiation (9 Gy) of animals. Levels of PGE2in extracts from proximal jejunum of mice was estimated using PGE2 ELISA Kit (Abcam, Cat no. 133021) at different time points after sham irradiation or post-irradiation with 9 Gy with or without G-003M pre-treatment.

### Cytokine estimation in mice serum

Blood samples were collected at different time-points post treatment (6hr, 24hr) via cardiac puncture in mice from different treatment groups. Briefly, blood samples were centrifuged at 6000xg for 10 min at 4°C. Serum was aliqouted and stored at -80°C until analysis. Levels of various cytokines (IL-6, TNF-α, G-CSF) in mice serum were determined by using respective BD^TM^ Cytometric Bead Array (CBA) Flex Set (BD Biosciences, USA) on dual laser flow analyzer (LSRII, Becton Dickinson Biosciences, USA) according to manufacturer’s instructions.

### Apoptosis detection by AnnexinV FITC-PI dual staining

Validation of our apoptosis study was done by using AnnexinV-PI dual staining followed by flow cytometric measurements. For this assay, manufacturer’s protocol mentioned in Apoptosis Detection Kit (Sigma, Cat no. APOAF) was followed. Briefly, IEC were isolated as described above and washed twice with DPBS. Cells were resuspended in 1x Binding Buffer at a concentration of 1x10^6^cells/ml and 500μL of the apoptotic cell suspension was added to 12x75 mm test tube. 5μL of Annexin V FITC Conjugate and 10 μL of Propidium Iodide Solution was added to each cell suspension and incubated at dark for 10 min at room temperature. Fluorescence of the cells was immediately determined by dual laser flow analyzer (LSRII, Becton Dickinson Biosciences, USA).

### Terminal deoxynucleotidyl transferase dUTP nick end labeling (TUNEL) assay in mice intestine

TUNEL assay was performed on jejuna intestinal sections using TUNEL Apoptosis Detection Kit (Millipore, Billerica, MA, Cat no. 17–141) as per manufacturer’s instruction. Briefly, sections were depraffinized and treated with proteinase K. The sections were incubated with terminal deoxynucleotidyl transferase (TdT) end-labeling cocktail, stained with Avidin-FITC and counterstained with 4′,6′-diamidino-2-phenylindole (DAPI). Stained sections were visualized under upright epifluorescence microscope (BX63, Olympus, Japan) and images were captured at 40X microscopic magnification for quantification of TUNEL positive cells per crypt. Representative images captured at 400x magnification are presented in results.

### Histology

For histological analysis, the jejunum region of gastrointestinal tissue was excised from mice of different treatment groups and subsequently fixed in 10% buffered formalin and processed for histological examination. Tissue sections (4–5 μm thick) embedded in paraffin wax, were cut on semiautomatic microtome (Spencers, India) and stained with hematoxylin and eosin (H & E) dye. All the samples were examined for histological features of gastro-intestinal damage on a light compound microscope (BX50, Olympus, Japan). Representative images captured at 20X magnification are presented in results.

### Crypt survival assay

Surviving crypts were measured in 6 to 8 weeks old mice sacrificed at 3.5 days after 9 Gy exposure with or without G-003M treatment. Each mouse was injected a single dose of 120 mg/kg BrdU (Sigma-Aldrich, Cat no. B5002) intraperitoneally 2hr before sacrifice to label the S phase cells. 5 μm paraffin sections were prepared from proximal jejunum. The viability of each surviving crypt was confirmed by immuno-histochemical detection of BrdU incorporation into five or more epithelial cells within each regenerative crypt by BrdU IHC kit (Millipore, Cat no. 2760). BrdU-positive cells were scored in 100 crypts per mouse, with a minimum of 3 mice per group. Representative images captured at 20X magnification are presented in results. Data were reported as mean ± SD.

### Tumor mice model

Swiss Albino ‘strain A’ (6–8 weeks old) mice maintained with a natural day/night cycle at the Institute’s experimental animal facility were used in the study. The tumor used was immunogenic Ehrlich ascites tumor (EAT) cells (strain F-3) obtained from Institute for Biophysics, University of Frankfurt, Germany and maintained by serial passage of tumor cell suspension in the peritoneal cavity of strain ‘A’ mice which forms solid tumors, when injected subcutaneously. EAT cells for initiation of subcutaneous tumors was obtained in ascitic form from the peritoneal cavity of Swiss Albino strain ‘A’ mice that had been serially passaged and maintained with Ehrlich ascites tumor (EAT) cells. Subcutaneous tumors were developed by injecting 5~9x10^6^ viable tumor cells under the skin on the right hind leg. Eight to 10 days after implantation, when the tumors reached approximately 100 mm^3^ in volume the mice (3–6 per group) were randomised by weight into five experimental groups. The five groups consist of (I) Untreated (mice with no tumor and no treatment) (II) Tumour only (mice with tumor but no treatment) (III) G-003M (tumor bearing mice treated with G-003M (6.5 mg/kg body wt) alone i.m.) (IV) IR (tumor bearing mice treated with focal irradiation of 10 Gy) (V) G-003M+IR (tumor bearing mice treated with intramuscular injection of G-003M followed by focal irradiation of 10 Gy to the tumor). The tumors were subjected to four consecutive treatments of 2.5 Gy localised to the tumor area in rear dorsum (a cumulative dose of 10 Gy) at a dose rate 1.8–2.5 Gy/min ^60^Co source cobalt teletherapy unit (Cobalt Tele Therapy, Bhabatron II).

### Tumor volume measurement

In order to determine tumor volume by external caliper, tumor growth was followed by measuring three mutually orthogonal tumor diameters (*e*1, *e*2, *e*3) with vernier calliper on each consecutive day. Tumor volume based on caliper measurements was calculated by the formula:

V=π×e1×e2×e3/6

### Haematological assay in tumor bearing mice

Blood samples were collected from orbital plexus of differentially treated groups in 2 ml heparinised vacutainers. Total leukocyte count (TLC), red blood cells (RBC), haemoglobin from the collected blood was analyzed on fully automated five part haematology analyser (Siemens, Advia 2220). Data are represented as mean ± SD of values retrieved from six mice individually.

### Statistical analysis

All results are represented as the mean ± SD of three independent experiments. The 30-day survival data was plotted using Kaplan-Meier analysis. Significant differences between different treatment groups was established by either the Student’s *t*-test or one-way analysis of variance (ANOVA) using the Student-Newman-Keuls Method of pair wise multiple comparisons. Values of *p<*0.05 were considered statistically significant. For each analysis, experimental unit was an individual animal.

## Results

### G-003M protects C57BL/6J mice against whole body lethal irradiation

**Fig 1:**
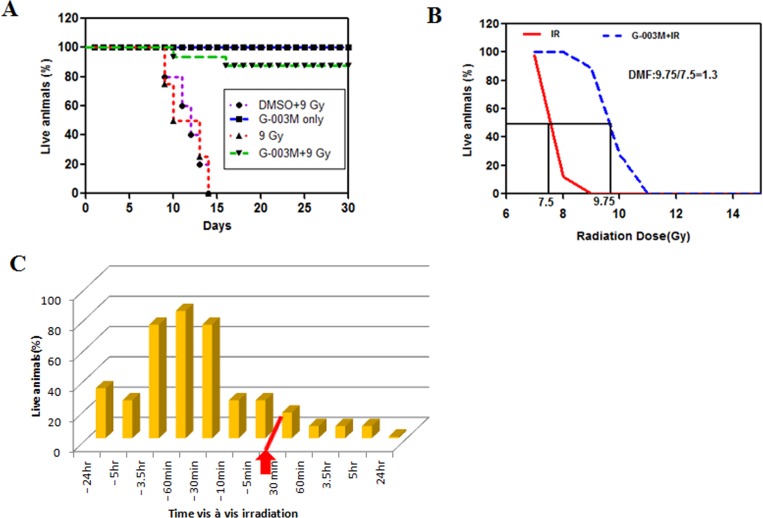
G-003M mediated radioprotection in C57BL/6J mice. (A) Groups of C57BL/6J (n = 18) mice were injected with G-003M or DMSO 60 min before 9 Gy total body γ-irradiation (TBI). Representative result from one of three independent experiments is shown. The difference in survival between untreated and treated groups was statistically significant on days 15 to 30 after 9 Gy TBI. (B) Estimation of dose modify factor (DMF) of G-003M. The ratio of LD 50 doses of radiation treatment with/without G-003M gives DMF of 1.3. (C) Groups of C57BL/6J mice (n = 12) were injected with G-003M (6.5 mg/kg) at the indicated time intervals relative to 9 Gy TBI (red arrow). The percent survival of mice at day 30 post irradiation is plotted.

To explore the *in vivo* radio protective potential, G-003M was injected at 6.5mg/kg through intramuscular route in C57BL/6J mice 60min before total body γ-irradiation (TBI). Upon 9 Gy irradiation in C57BL/6J mice (n = 18), gradual loss in body weight, food and water intake, ruffled fur was observed and consequently all the mice died within 15 days of exposure (Fig 1A). Treatment with G-003M delivered (89±6.9)% protection in lethally (9 Gy) irradiated mice. Initially, fall in the body weight was observed in this group but recovered later and within 30 days weight of all the surviving mice was corresponding to untreated animals. DMSO alone did not provide any survival benefits to lethally (9 Gy) irradiated mice. G-003M alone did not exert any toxicity or mortality at its effective dose (Fig 1A). We next investigated the time frame for effective administration of G-003M at different time intervals pre or post irradiation. G-003M delivered protection to mice against high dose (9 Gy) of radiation only when administered between 30 min to 3.5 hr before TBI (Fig 1C). Our drug did not provide any survival benefits to mice if injected before this time interval or after irradiation. In all the studies reported here, 60 min was the standard time interval between drug treatment and radiation exposure.

### G-003M reduces radiation induced ROS levels in intestinal epithelial cells (IEC)

**Fig 2:**
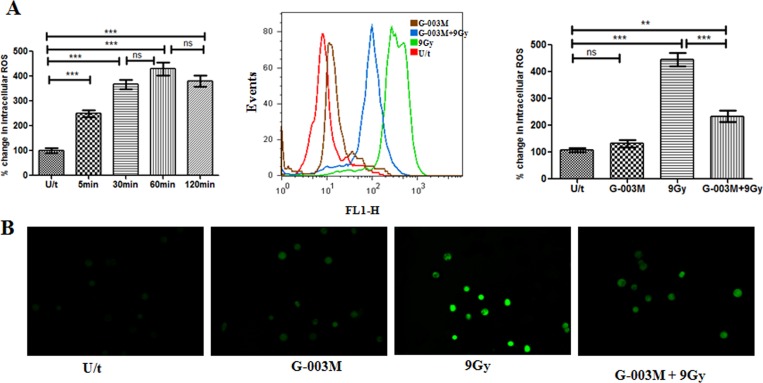
G-003M reduces lethal radiation induced ROS level in mice intestinal epithelial cells (IEC). (A) Flow cytometry histogram showing changes in the ROS levels of IEC in mice (n = 3) that were left untreated (U/t), given G-003M without 9Gy radiation (G-003M), or exposed to 9 Gy TBI 60 min after injection of DMSO (9 Gy) or G-003M (G-003M+9 Gy). Bar diagram showing % change in intracellular ROS production at different time intervals post irradiation compared to untreated mice and in different treatment groups is also shown. (B) Fluorescent microscope (magnification; 40X) imaging of ROS levels in IEC was observed in mice injected with G-003M or DMSO 60 min before 9 Gy TBI. The ROS levels were measured in IEC after 1 hr of treatment by staining with 2',7'-dichlorofluorescein diacetate (DCF-DA) dye. All the figures are representative of three independent experiments and expressed as mean± SD. A value of p<0.05 is considered statistically significant. ns = nonsignificant, * = p < 0.05, ** = p < 0.01, *** = p < 0.001.

Upon irradiation, ROS generation in mice IEC was measured at different time intervals. The level of these reactive oxygen species was found enhanced within 5 min of 9 Gy exposure and maximum was evident at 1 hr however, the same continued upto 2 hr post irradiation. As shown in (Fig 2A), irradiation (9Gy) caused 4 fold increase (p<0.001) in ROS production as compared to sham irradiated control mice. Single dose intramuscular administration of G-003M at its therapeutic concentration, 1hr prior to radiation, reduced the ROS generation by 2 fold (p<0.001) when compared with corresponding irradiated group mice (Fig 2A). In addition, intensity of ROS generation in DCF-DA stained IEC of differentially treated mice at 1 hr was analyzed using fluorescent microscopy. In concurrence with the flow analysis, significant reduction (p<0.001) of ROS generation was evident in the G-003M pretreated IEC as compared to irradiated IEC(Fig 2B). The above observations clearly demonstrate the ROS scavenging ability of G-003M in mice IEC.

### G-003M downregulates radiation induced activation of Nuclear factor-ĸB (NF-κB) and its effectors COX-2 and iNOS

**Fig 3:**
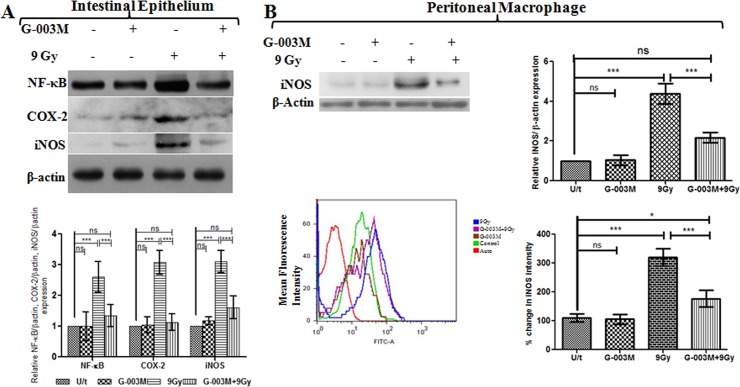
G-003M inhibits NF-κB activation and its target effectors COX-2, iNOS in mice jejunum exposed to γ-irradiation (TBI). (A) G-003M was administered 1 hr prior to 9 Gy dose of gamma radiation and protein extracts (30 μg) were loaded onto 8–12% SDS-polyacrylamide gel, electrophoresed and transferred to nitrocellulose membrane and probed with NF-κB, COX-2, iNOS antibody. Quantification of NF-κB, COX-2 and iNOS expression was normalised to β-actin expression, using IMAGE lab software for densitometry. (B) Western blot and flow cytometry analysis of iNOS expression in mice peritoneal macrophages at 24 hr showed downregulation of iNOS expression upon G-003M treatment. All the figures are representative of three independent experiments and expressed as mean± SD. A value of p<0.05 is considered statistically significant. ns = nonsignificant, * = p < 0.05, ** = p < 0.01, *** = p < 0.001.

As NF-κB plays a critical role in regulating radiation-induced inflammatory and immune responses, modulation of NF-κB expression and its effector proteins, COX-2 and iNOS by G-003M was assessed using western blot assay. The analysis indicated > 2 fold (p<0.001) increase in the expression of total NF-κB (p65 subunit) in the mice jejuna at 6 hr post exposure to 9 Gy radiation (Fig 3A). Compared to irradiation, administration of G-003M exhibited down expression (p<0.001) of NF-κB at same time interval. G-003M treatment alone did not induce any alteration in NF-κB expression (Fig 3A). The expression of NF-κB target proteins COX-2 and iNOS in the jejuna was also examined in irradiated and G-003M pre-treated mice. Following 9Gy whole body irradiation, COX-2 was found >2.5 fold increased (p<0.001) at 6 hr of exposure while iNOS got enhanced by ~ 3 fold (p<0.001) when compared with control group. G-003M administration resulted in significant downregulation of both COX-2 and iNOS expression. No significant change in the expression of these proteins was observed in G-003M (alone) group (Fig 3A).

Additionally, the expression of iNOS was also examined in peritoneal macrophages by western blotting and flow cytometry in differentially treated mice (Fig 3B). Western blot analysis demonstrated up regulation of iNOS expression by 4 fold (p<0.001) in irradiated macrophages at 24 hr following 9 Gy irradiation. The same was found significantly (p< 0.001) down regulated by G-003M pre-treatment (Fig 3B). In concurrence with the western blot analysis, flow cytometric assay also revealed the anti-inflammatory property of G-003M through significant reduction (p<0.001) of iNOS expression in G-003M pre-treated macrophages as compared to the 9 Gy irradiated group at 24 hr time interval (Fig 3B). These findings clearly indicate that G-003M has minimized the damaging effect of radiation by suppressing NF-κB transcription factor activation and its effector proteins.

### G-003M restricts nuclear translocation of NF-κB in irradiated mice IEC

**Fig 4:**
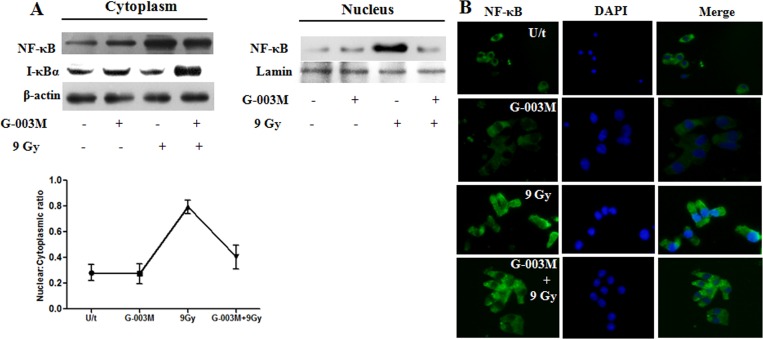
G-003M restricts NF-κB nuclear translocation in mice jejunum exposed to gamma irradiation. (A)G-003M was administered intra-muscularly into mice 1 hr prior to 9Gy whole body irradiation. After 6 hr, mice were sacrificed and intestinal epithelial nuclear and cytoplasmic protein extracts were prepared and assayed by immunoblotting for NF-κB, I-κBα expression. (B) Immunoflourescence (magnification; 40X) staining in intestinal epithelial cells (IEC) for NF-κB translocation was observed at 6 hr post-irradiation in mice injected with G-003M or DMSO 60 min before 9 Gy TBI. Corresponding nuclear: cytoplasmic ratios of NF-κB localisation in unstimulated control mice and 6 hr post irradiated (9 Gy) mice and mice pre-treated G-003M formulation 1 hr prior to 9 Gy whole body gamma radiation was quantified by IMAGE J software and expressed as mean±SD. 200 cells were randomly selected for scoring per animal. All the figures are representative results of three independent experiments.

To explore the possibility whether the inhibitory effect of G-003M on NF-κB was due to its suppression of IκBa degradation via phosphorylation, the cytoplasmic level of IκBa was determined by western blot analysis. G-003M pre-treatment significantly (p< 0.001) inhibited the phosphorylation and degradation of IκBa at 6 hr time-point post-irradiation in mice gut epithelium, following lethal dose (9Gy) of γ-irradiation (Fig 4A). The level of p65 subunit in the nuclear and cytoplasmic sub fractions of intestinal epithelial cells was determined by western blot analysis. Upon irradiation the expression of p65 in the nucleus increased by 3.5 fold (p<0.001), which was subsequently blocked by G-003M pre-treatment (Fig 4A). In agreement with the western blot results, the immunoflourescence studies also indicated increased expression of NF- ĸB (p65 subunit) in the nuclei of irradiated IEC, while administration of G-003M 60 min before radiation resulted in reduced (p< 0.001) translocation of p65 to the nucleus (Fig 4B).These findings clearly demonstrate that the inhibitory role of G-003M on NF-κB nuclear translocation is exhibited through upregulation of IκBa expression and blockade of its phosphorylation and degradation in lethally irradiated mice epithelium.

### G-003M reduces radiation induced nitric oxide (NO) production in intestinal epithelial cells and peritoneal macrophages

**Fig 5:**
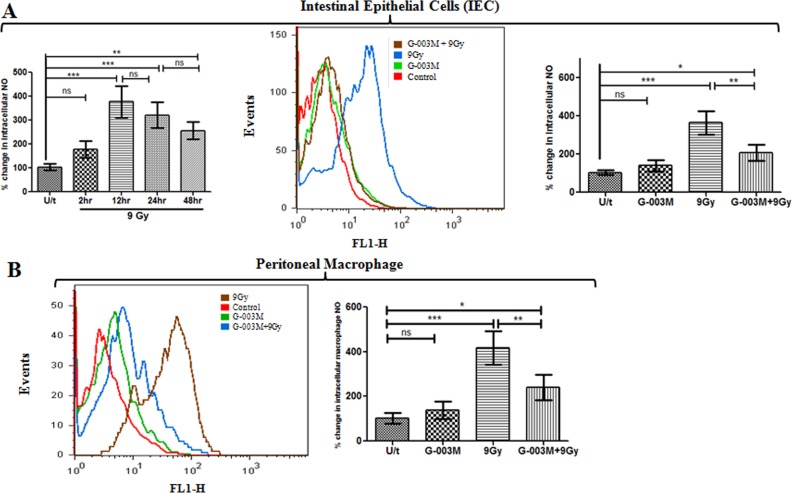
G-003M blocked nitric oxide (NO) generation in intestinal epithelial cells (IEC) and peritoneal macrophages. (A) Flow cytometry histogram showing changes in NO levels in IEC of mice (n = 3) that were left untreated (U/t), G-003M alone, exposed to 9 Gy TBI 60 min after injection of DMSO (9 Gy) or G-003M (G-003M+9Gy). Bar diagram showing % change in intracellular NO production at different time intervals post irradiation compared to untreated mice and in different treatment groups is also shown. (B) Depicts NO levels in peritoneal macrophages after exposure to 9 Gy TBI with or without G-003M treatment. Quantification of NO level is presented as percent change in mean fluorescence intensity of different treatment groups. Cells from untreated mice were used as controls. All the figures are representative results of three independent experiments and expressed as mean±SD. A value of p<0.05 is considered statistically significant. ns = nonsignificant, * = p < 0.05, ** = p < 0.01, *** = p < 0.001.

Upon exposure to 9 Gy radiation, levels of intracellular nitric oxide (NO) in the IEC were found nearly 1 fold enhanced in comparison to control group within 2 hr of exposure. At 12 hr this increase was maximum and almost >3.5 fold (p<0.001) higher than control. However, NO levels started decreasing at later time points of study i.e. at 24 hr increase was around >2.5 fold and at 48 hr it was only 2 fold when compared with the untreated control (Fig 5A). Since 12 hr had shown maximum increase in NO levels in the radiation group, drug treatment was restricted to this time point only. G-003M pre-treatment significantly inhibited radiation induced NO levels by 45% (p<0.01) at 12 hr of study compared to corresponding radiation only group. G-003M (alone) administration resulted in marginal (non-significant) elevation of NO level when compared to control group (Fig 5A).

In the peritoneal macrophages, upon 9 Gy exposure intracellular NO generation increased significantly by 4 fold (p<0.001) at 24 hr as compared to the untreated group (Fig 5B). However, this increase was significantly attenuated by G-003M pre-administration and the NO level was found decreased by 2 fold (p<0.001) as compared to radiation only group but remained elevated by 1.5 fold in comparison to untreated control group (Fig 5B).

### G-003M blocks Prostaglandin E2 (PGE2) production in lethally irradiated mice jejunum

**Fig 6:**
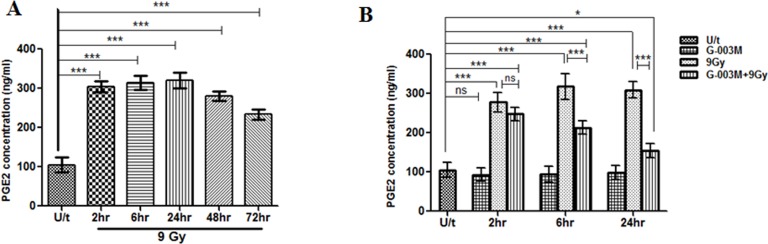
G-003M blocked prostaglandin E2 (PGE2) production in mice jejunum. (A) PGE2 generation was measured by using PGE2 ELISA Kit at different time intervals following 9 Gy TBI in C57BL/6J mice jejunum. (B) G-003M pre-treatment suppressed the production of PGE2 (ng/ml) at 2 hr, 6 hr and 24 hr time intervals post-irradiation. Each data point represents mean of triplicate±SD. ns = nonsignificant, * = p < 0.05, ** = p < 0.01, *** = p < 0.001.

Following 9 Gy irradiation in C57BL/6J mice (Fig 6A), levels of PGE2 in jejunum tissue extracts increased by 3 fold (305ng/ml) than baseline values (100 ng/ml) within 2 hr post-irradiation and the increase was sustained upto 24 hr. At 48 hr of the study marginal fall in PGE2 levels in the same group mice was noticed and the levels further declined though not significantly at 72 hr post exposure. G-003M pre-treatment 1 hr prior to radiation inhibited PGE2 synthesis marginally (Fig 6B) at 2hr time point of study as compared to corresponding radiation only group. However, in the same group, radiation mediated increase in PGE2 levels was found significantly (p<0.001) reduced at 6 hr and 24 hr (Fig 6B). PGE2 levels in G-003M (alone) treated group was found comparable to the controls at all the studied time-points.

### G-003M regulates cytokine level in response to γ-irradiation

**Fig 7:**
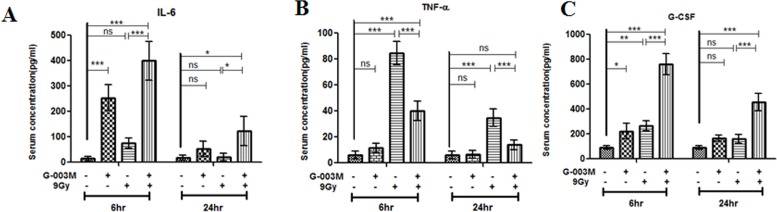
G-003M regulates cytokines (IL-6, TNF-α and G-CSF) levels. After isolation of blood serum at different time points (6 hr, 24 hr) following 9 Gy γ-irradiation, serum levels of (A) IL-6 (B) TNF-α and (C) G-CSF cytokines (pg/ml) in different treatment groups was determined by flow cytometry using respective BD^TM^ Cytometric Bead Array (CBA) Flex Set. Each data point represents mean of triplicate ± SD. ns = nonsignificant, * = p < 0.05, ** = p < 0.01, *** = p < 0.001.

The serum level of cytokines TNF-α, IL-6 and G-CSF were measured in irradiated and G-003M pre-treated C57BL/6J groups at 6 hr and 24 hr by flow cytometry. Serum levels of IL-6 increased after G-003M (alone) injection, peaked at 6 hr post-dosage by 130~140 folds higher than the 3–4 pg/ml baseline level and normalised by 24 hr of treatment. A mild increase (non-significant) in IL-6 level was found in the serum following exposure to 9 Gy radiation at 6 hr time interval. However, in the G-003M pre-treated and irradiated C57BL/6J group, the serum levels of IL-6 was found induced by 5 fold (p<0.001) as compared to corresponding radiation only group at the same time-interval. A modest induction (non-significant) in serum levels of IL-6 was evident at 24 hr following 9Gy radiation. G-003M pre-treatment resulted in 6 fold (p<0.001) increase in IL-6 level as compared to corresponding lethally irradiated group (Fig 7A).

At 6 hr time-interval following 9Gy exposure, TNF-α level was found enhanced by 13~14 folds in the serum of the irradiated group as compared to control. This increase in serum TNF-α level was consequently countered by G-003M treatment, resulting in reduction of TNF-α levels by 2 fold (p < 0.001) as compared to lethally irradiated mice group. By 24 hr, serum levels of TNF-α in the irradiated group declined by 55% as compared to 6 hr but remained elevated by 5.5 fold (p<0.001) as compared to sham-irradiated control mice. G-003M treatment resulted in further decrease of serum TNF-α by ~60% as compared to irradiated group (Fig 7B). G-003M (alone) did not cause any significant alteration of TNF-α level at both the time-points of study compared to untreated control groups.

Following 9 Gy TBI, serum G-CSF levels increased by 2 fold (100 pg/ml baseline level) at 6 hr compared to control group and it normalised to the baseline level by 24 hr post-irradiation. At 6 hr, serum levels of G-CSF increased by 2 fold in G-003M pre-treated mice as compared to irradiated mice. Levels of G-CSF declined by 40% in G-003M pre-treated mice at 24 hr but remained elevated by 2.5 fold as compared to corresponding irradiated group (Fig 7C). G-003M (alone) induced a 2.4 fold increase in serum levels of G-CSF at 6 hr compared to the control groups, however, it restored to baseline level by 24 hr post-treatment.

### G-003M inhibits intestinal apoptosis caused by radiation

**Fig 8:**
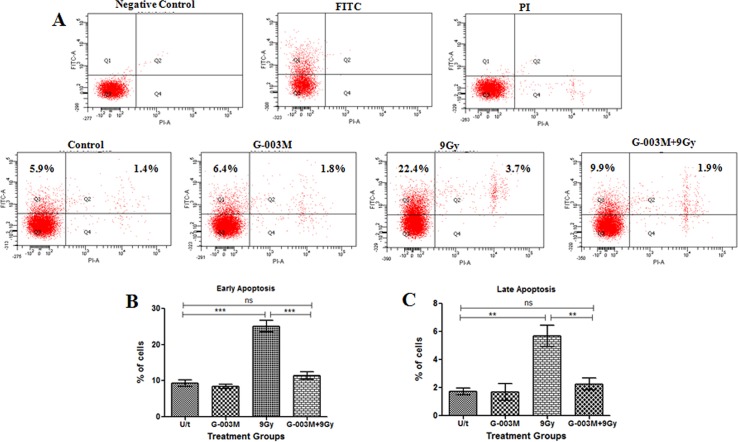
G-003M attenuates radiation-induced apoptosis in mice IEC. (A) IEC were collected from different treatment groups at 6 hr after irradiation, stained with propidium iodide and Annexin V-FITC and analyzed by flow cytometry. Representative diagrams of distribution of stained cells are shown. Bar graphs of (B) early apoptotic and (C) late apoptotic cells expressed as a percent of total cells for each treatment with SD from three independent experiments are shown. A value of p < 0.05 is considered statistically significant ns = nonsignificant, * = p < 0.05, ** = p < 0.01, *** = p < 0.001.

**Fig 9:**
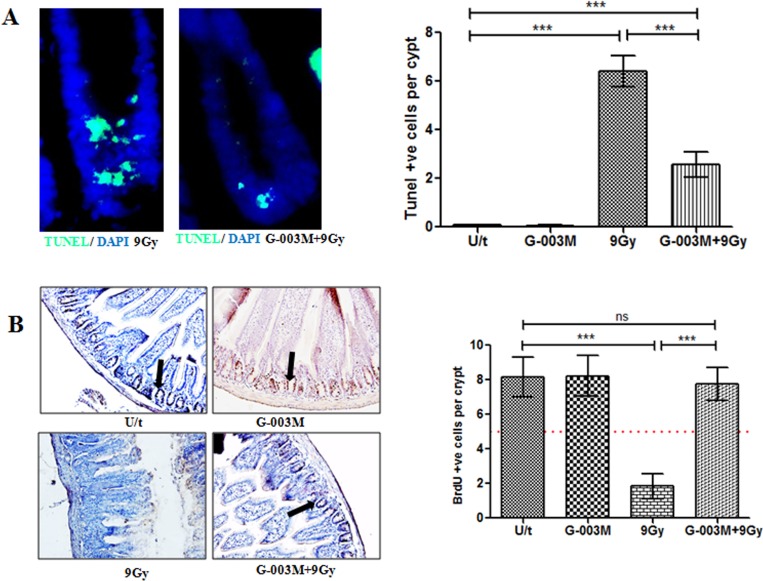
G-003M protects mice GI from radiation injury. (A) Terminal deoxynucleotidyl transferase–mediated deoxyuridine triphosphate nick end labeling (TUNEL) staining was performed in C57BL/6J mice jejunum using TUNEL Apoptosis Detection Kit at 4 hr post-irradiation. Proximal jejunum was taken from mice injected with G-003M or DMSO 60 min before 9 Gy TBI for TUNEL staining. Green flourescence indicates apoptotic cells. Nuclei of cells were stained with 4′, 6′-diamidino-2-phenylindole (DAPI, blue). (B) In vivo immunohistochemical detection of S phase cells incorporating BrdU (black arrowhead) in the crypts of the small intestine. BrdU-positive cells of differentially treated groups were scored in 12 complete, longitudinal sections for each animal. Dashed red line: number of BrdU-positive cells considered critical for crypt survival. Each data point represents mean of triplicates ± SD. A value of p < 0.05 is considered statistically significant. ns = nonsignificant, * = p < 0.05, ** = p < 0.01, *** = p < 0.001.

To evaluate intestinal epithelial cell apoptosis, Annexin-PI dual staining assay was performed (Fig 8A). Compared to the control, IEC isolated from the irradiated mice (9 Gy) showed a significant increase in the number of early apoptotic cells after 6 hr of exposure (5.9% vs. 22.4%), and for late apoptosis the values were (1.4% vs. 3.7%) in control vs. radiation only group (Fig 8A). In the group that had been pre-treated with G-003M, the number of apoptotic cells was found markedly decreased in comparison to the radiation treatment group (9.9% vs. 22.4% for early apoptosis and 1.9% vs. 3.7% for late apoptosis) (Fig 8B and 8C). No alteration in the number of apoptotic cells was observed in IEC of the mice treated with G-003M alone when compared with control group. TUNEL staining in the jejuna epithelium of irradiated mice showed a significant > 6 fold (p < 0.001) increase in TUNEL-positive cells (TPC) per crypt region as compared to sham-irradiated control (Fig 9A). Interestingly, G-003M pre-treatment resulted in significant reduction > 55% of TUNEL-positive cells when compared to the lethally irradiated group.

### G-003M enhances crypt survival after radiation

To determine G-003M mediated regulation of crypt stem cell survival in response to radiation (9 Gy) damage, we treated C57BL/6J mice with or without G-003M prior to γ-irradiation and examined crypt stem cell survival at 3.5 days by 5-bromo-2'-deoxyuridine (BrdU) incorporation into proliferating crypt cells of the jejunum, using modified microcolony assay. After 9 Gy, the number of viable crypts per jejuna circumference decreased by 4.4 fold (p < 0.001) as compared to control mice (Fig 9B). However, G-003M pre-treatment resulted in significant enhancement of crypt survival by 75% (p < 0.001) in comparison to corresponding irradiated group.

### G-003M negatively regulates p53 signaling pathway in mice intestinal epithelium

**Fig 10:**
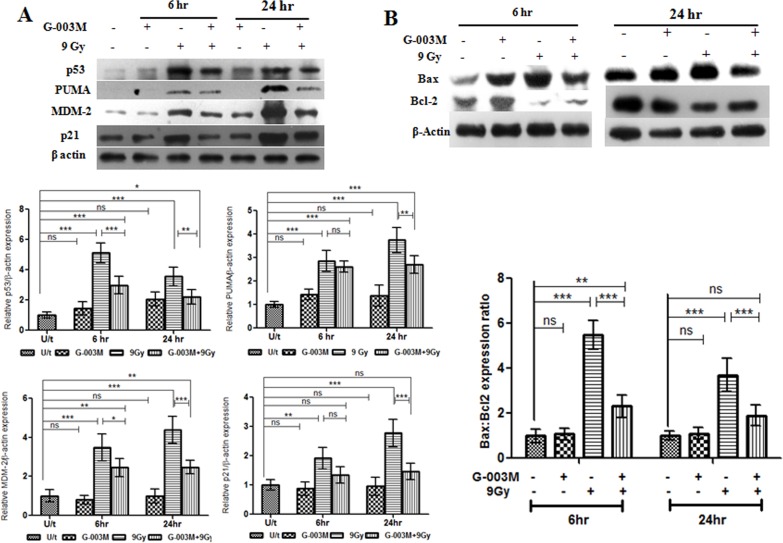
G-003M negatively regulates p53 signaling pathways and bax, bcl2 expression ratio in mice jejunum. (A) G-003M was administered 60 min prior to 9 Gy dose of gamma radiation and protein extracts (30 μg) were loaded onto 12% SDS-polyacrylamide gel, electrophoresed and transferred to nitrocellulose membrane and probed with p53, PUMA, MDM-2, p21 antibody. The western blot is representative results of three independent experiments. Quantification of p53, PUMA, MDM-2, p21 expression was normalised to β-actin expression, using IMAGE lab software for densitometry. (B) G-003M treatment resulted in up regulation of bcl2 and downregulation of bax expression at both 6 hr and 24 hr post-irradiation in mice jejunum as evident by western blot analysis. Each data point represents mean of triplicates ± SD. A value of p < 0.05 is considered statistically significant. ns = nonsignificant, * = p < 0.05, ** = p < 0.01, *** = p < 0.001.

To understand the possible mechanism by which G-003M inhibits radiation-induced apoptosis, we examined the expression of p53, PUMA, MDM-2 and p21 in the mice intestinal epithelium. In mice exposed to 9 Gy γ-irradiation expression of pro-apoptotic p53 and its target PUMA increased significantly by 5 fold and 2.5 fold respectively at 6 hr when compared to untreated control group (Fig 10A). This radiation induced expression of p53 and PUMA in the gut epithelium was countered by 1.5 fold (p < 0.001) and 1 fold (non-significant) upon G-003M administration. By 24 hr of 9 Gy exposure, expression of p53 and PUMA was found enhanced by 3.5 fold compared to the untreated mice. G-003M treatment resulted in further downregulation of p53 and PUMA by 1.5 fold (p < 0.01) and 1.3 fold (p < 0.01) respectively. G-003M could also restore the radiation induced p21 and MDM-2 expression in the small intestine to the basal level. No significant variation in the expression pattern of p53, PUMA, MDM-2 and p21 was found in G-003M (alone) group at all the time-points of study (Fig 10A). Thus, G-003M inhibits radiation induced apoptosis by negatively regulating p53 and PUMA expression in the mice intestine.

### G-003M modulates Bax, Bcl2 expression in lethally irradiated mice jejunum

We examined the effect of G-003M pre-treatment on Bax and Bcl2 expression in lethally irradiated mice jejunum. Expression of Bax enhanced significantly (> 2 fold; p < 0.001) at 6 hr of 9 Gy exposure and the increase was evident upto 24 hr as compared to sham-irradiated control mice. On the other hand, γ-irradiation resulted in downregulation of Bcl2 expression from 6 hr upto 24 hr of exposure in mice intestine. The radio protective effect of G-003M treatment in mice intestine was evaluated at both 6 hr and 24 hr time–intervals post radiation exposure. Interestingly, G-003M administration resulted in strong suppression of Bax expression (p < 0.001) and subsequent enhancement in Bcl2 expression (p < 0.001) at both the time intervals, finally leading to significant reduction (p < 0.001) of Bax/Bcl2 expression ratio in mice jejunum as evident by western blot analysis (Fig 10B). However, G-003M alone did not induce any significant change in Bax/Bcl2 expression ratio at both the time intervals. Thus, G-003M induces an anti-apoptotic response by reducing the Bax/Bcl2 expression ratio in mice intestine.

### G-003M restores radiation induced alterations in jejuna morphology

**Fig 11:**
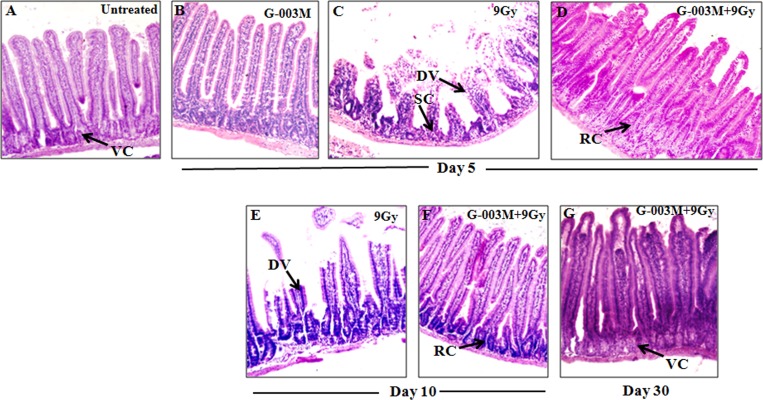
G-003M treatment minimised radiation-induced histological alterations during intestinal injury. H&E-stained sections of C57BL/6J jejunum obtained at (B) (C) (D) 5^th^ day, (E) (F) 10^th^ day and (G) 30^th^ day after exposure to 9 Gy TBI with or without G-003M pretreatment. Representative micrographs were taken at 20X magnification.

The irradiated jejunum of C57BL/6J mice showed symptoms of severe damage after day 5 and day 10 of lethal radiation exposure (9 Gy). Increased presence of non-viable crypts, disrupted epithelial lining, denuded and fused villi, decrease in mean crypt-villus height was observed in the longitudinal sections of jejunum when compared with that of untreated mice (Fig 11C and 11E). The histological features of the group that had been pretreated with G-003M (-1 hr) prior to 9 Gy exposure exhibited reduced jejuna damages as the mucosal and sub mucosal layers were found relatively intact at both day 5 and day 10 of the study. The crypts were viable and better organized compared to irradiated group. Although, villi were seen marginally reduced in size at day 5 post treatment but most of them were found intact by day 10 (Fig 11D and 11F). And by day 30, G-003M could completely restore the jejuna morphology due to presence of significant number of viable crypts, intact villi and increase in crypt-villus height (Fig 11G).

### G-003M did not reduce the sensitivity of tumor tissue to γ-irradiation

**Fig 12:**
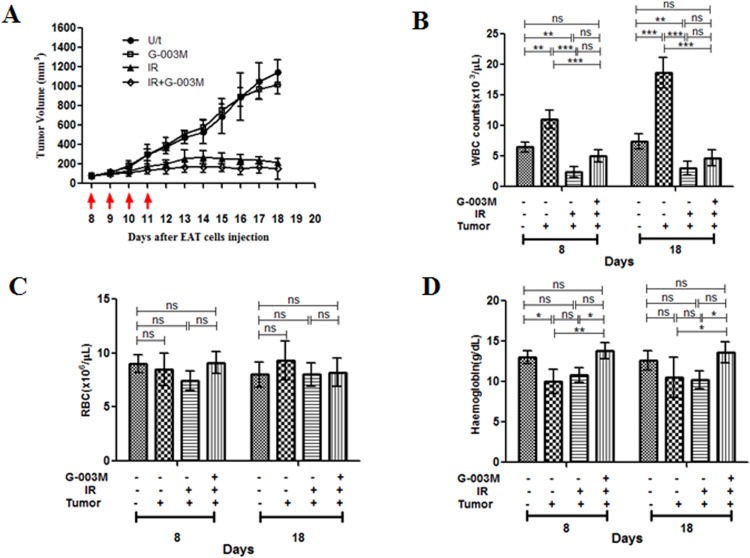
**G-003M does not reduce the radio sensitivity of tumor in mice** (A) Swiss albino ‘strain A’ mice carrying EAT tumors (6 mice/group) were treated with DMSO alone (U/t, untreated control), radiation alone (IR, 2.5 Gy localised radiation per fraction), G-003M alone (6.5 mg/kg i.m. per injection) or radiation together with G-003M (IR+G-003M). All treatments were applied on days 8, 9, 10 and 11 after EAT cell injection (red arrows). Tumor volume was measured every consecutive day. Values are medians of tumor volumes with error bars indicating S.D. Variation in tumor growth rate among groups were analyzed by two-way repeated measures ANOVA and by Student’s t test (two-tailed, unequal variances). Estimation of (B) WBC (C) RBC and (D) Hemoglobin in tumor bearing mice. The blood components were measured at 8^th^ and 18^th^ day in different treatment groups. The counts between irradiated and G-003M treated groups were statistically significant. A value of p < 0.05 is considered statistically significant. ns = nonsignificant, * = p < 0.05, ** = p < 0.01, *** = p < 0.001.

To assess the differential radio protective potential of G-003M in normal vs. tumor tissues during anticancer radiotherapy procedure, we developed a model in which tumor-bearing mice were subjected to four consecutive treatments of 2.5 Gy localised to the tumor area in rear dorsum (a cumulative dose of 10 Gy). To evaluate whether G-003M affected the radio sensitivity of the tumors, groups of the mice were injected with G-003M 1 hr before each radiation treatment. The model used was Ehrlich ascites tumor (EAT) cells implanted in Swiss Albino ‘strain A’ mice and grown subcutaneously. The antitumor effect of focal radiation was measured in the form of significant delay (p < 0.001) in tumor growth as compared to untreated control (Fig 12A). The combined treatment of G-003M and focal radiation had no effect on the tumor regression caused by radiation.

In the focally irradiated tumor mice, depletion of white blood cells (WBC) was evident at the 8^th^ and 18^th^ day of the study when compared to tumor untreated mice group. This fall in WBC counts was successfully restored by G-003M pre-treatment and a consistent increase in WBC counts was evident at both the time-points. By 18^th^ day, the WBC counts were almost similar to normal control groups. In the groups where tumor bearing mice were neither irradiated nor treated with G-003M, WBC count was found 2.5 fold higher (p < 0.001) than untreated control mice (Fig 12B). Unlike the WBC levels, exposure to 10 Gy focal radiation did not significantly alter the red blood cells (RBC) and haemoglobin levels in the tumor mice model (Fig 12C and 12D) at both 8^th^ and 18^th^ day of study. Levels of haemoglobin was found significantly enhanced (p < 0.05) in the G-003M treated mice when compared to irradiated tumor group at 8^th^ day of the treatment. By 18^th^ day, haemoglobin levels in the drug treated tumor mice were comparable to those of untreated control mice (Fig 12D).

## Discussion

Rising apprehension over the possible exposure of humans to radiation has raised the need to develop countermeasures against radiation injuries. Unlike the haematopoietic injury which can be subsequently taken care by administering growth factors, bone marrow transplantation, blood transfusion etc. there is no approved medical countermeasure to recover from radiation induced GI damage.

Our thirty day survival study in mice has confirmed that G-003M could extend nearly 88% survival against lethal dose (9 Gy) of total body γ- radiation (TBI) (Fig 1A). The dose modifying factor (DMF) of G-003M in mice using whole body survival as end point was found to be 1.3, which is quite significant (Fig 1B). Though several other compounds are reported [11, 23] for similar or better DMF/survival benefits, their associated toxicity have limited their clinical use. Additionally, our formulation was found to retain its efficacy over a wide time frame (30 min to 3.5 hr) before radiation, which was a significant improvement over other antiradiation drugs studied so far. Amifostine, was effective only upto 30 min interval prior to irradiation [23], whereas other prophylactic agents like gamma-tocotrienol, pyridoxamine have shown maximum protection only when injected 24 hr before irradiation [24, 25].

Our studies have clearly demonstrated significant reduction in radiation induced intracellular accumulation of ROS in the mice IEC by G-003M (Fig 2). The reduction of ROS accumulation by G-003M has been majorly attributed due to the presence of rutin that has been revealed by our earlier studies [20]. Induction of antioxidant and cytoprotective enzymes, such as heme oxygenase-1 and SOD-1 through activation of the redox-sensitive transcription factor Nrf2 [20] might also be another possible reason in minimising oxidative stress in mice intestine.

Since intracellular ROS generation triggers the activation of NF-κB via the IKK signaling [26], we examined redox sensitive NF-κB expression and its target effectors responsible for initiation and maintenance of intestinal inflammation [27]. Immuno-blotting studies revealed negative regulation of NF-κB expression in intestinal tissue by G-003M (Fig 3A). Restricted translocation of p65 subunit from cytoplasm to nucleus of mice IECS was also evident in the G-003M treated group when compared to irradiated group. This might have been primarily achieved through G-003M mediated minimization in IκBα phosphorylation and also in its upregulated expression in the cytoplasm of lethally irradiated mice IECs (Fig 4). This observation was in resonance with curcumin that minimizes radiation induced acute inflammatory process in the intestine mainly through inhibition of NF-κB [28].

In response to pro-inflammatory stimuli, cyclooxygenase-2 (COX-2) and inducible isoform of nitric oxide synthase (iNOS) gets regulated by NF-κB driven trans-activation that mediates subsequent inflammation process [29]. COX-2 in turn enhances prostaglandin E2 (PGE2) synthesis that critically regulates crypt stem cell renewal [30]. Reports also suggest that increased iNOS expression and NO levels produced [31, 32] or its subsequent cytotoxic products [33] play a key role in the pathogenesis of chronic inflammation in the intestine [34, 35]. Thus agents that inhibit COX-2, iNOS expression and subsequent NO production can be a potential target for GI protection against radiation due to its anti-inflammatory role. In accordance, we also found that G-003M not only down regulated COX-2 expression but also reduced PGE2 production in the mice intestine (Figs 3A and 6). Similarly, G-003M pre-treatment also resulted in significant suppression of iNOS expression and NO production in both mice jejunum and peritoneal macrophages when compared to irradiated group (Figs 3 and 5). These observations support the anti-inflammatory effect of G-003M and its potential as radiation countermeasure. Earlier our group also reported significant iNOS inihibition in lung tissues of irradiated mice by our previously derived formulation [18]. In line with our observation, other agents like Aminoguanidine [36], 17-dimethylamino-ethylamino-17-demethoxygeldanamycin (17-DMAG) [37] have been reported for their radioprotective ability in intestine, blood and bone marrow of mice mainly through inhibition of iNOS.

As previously discussed, activation and translocation of Nuclear factor-κB (NF-κB) is a critical event linking inflammatory response with radiation injury. This process is accomplished mainly through NF-κB driven pro-inflammatory cytokines regulation that majorly contributes to tissue damage after radiation exposure [38]. Our studies showed lethal irradiation induced escalation of serum TNF-α level that contribute to DNA damage and cell death. However, this increase in serum TNF-α level was significantly blocked by G-003M pre-treatment when compared to the corresponding irradiated mice group. Suppression of serum TNF-α level clearly indicates the anti-inflammatory effect of our formulation. Interestingly, our study also demonstrated G-003M mediated enhancement of IL-6 levels in both non-irradiated as well as in irradiated animals. This increase in IL-6 levels probably supported the survival of bone marrow progenitor cells in the combined treatment group. In consonance to the reports reflecting G-CSF assisted proportional increase in survival, proliferation and differentiation of granulocyte progenitors [39], our data had also shown significant increase of serum G-CSF levels in G-003M pre-treated mice (Fig 7). G-003M mediated induction of IL-6 and G-CSF level is a critical event supporting the early recovery of hematopoietic system from lethal irradiation in mice.

We observed that 9 Gy of radiation exposure caused severe mucosal layer injury, including significant loss of viable crypt cells and disruption of villus integrity and functionality. These pathologic developments were barred significantly by G-003M, demonstrating its effective protection against TBI inflicted GI injury. This feature of G-003M was accomplished possibly by maintaining an intact intestinal epithelium which restored immune homeostasis through proper absorption of essential nutrients and boosted the overall recovery of mice GI system by enhancement in regeneration of crypt cells (Fig 11). Flow cytometric analysis also demonstrated effective inhibition of radiation-induced apoptosis in small intestinal epithelial cells by G-003M (Fig 8). Reports suggest that crypt apoptosis is blocked in either p53/PUMA null mice [40, 41]. Interestingly, our data also demonstrated p53 mediated PUMA suppression in response to G-003M pre-treatment in TBI mouse jejunum. Since p53-dependent PUMA induction is detrimental to the intestinal stem cells survival and subsequent regeneration of crypts, negative regulation of PUMA proved to be the predominant mechanism underlying intestinal radioprotection and crypt regeneration by our formulation (Fig 10). Results have also indicated that G-003M pre-treatment induces a cellular anti apoptotic signalling by reducing the expression of Bax/Bcl-2 in mice intestine.

Previous findings [42, 43] have reported certain compounds known for delivering differential protection. In parity, our data also illustrated the potential of G-003M in protecting normal tissues against the adverse side effects of focal radiotherapy procedure without compromising the radiation induced tumor regression. It was possible probably due to abnormal vasculature of the tumor tissue that restricted the absorption of G-003M at the tumor site or due to unknown response mechanism of the tumor cells. Increase in white blood cells (WBC) after day 8 and 18 of tumor induction (Fig 12B) clearly indicated the enhanced recruitment of different sub-sets of lymphocytes to the tumor site causing inflammation during cancer development [44]. Interestingly, depletion of WBC (lympho-depletion) in the combined treatment (G-003M+IR) group possibly assisted in resetting the homeostasis favouring reduction in inflammation. These findings indicate that though G-003M treatment leads to overall lympho-depletion, yet it restores normal peripheral blood counts required for anti-tumor response, which may contribute to cancer cure. However, further studies are warranted to identify key host factors for deciphering the mechanism of differential radioprotection of our formulation.

## Conclusions

In conclusion, our results have demonstrated the radio protective potential of G-003M and its mechanism of action in vivo. G-003M effectively prevented radiation induced inflammation and apoptosis in GI by negative regulation of NF-κB and p53 signaling pathways. Besides, it also stimulated granulocyte and megakaryocyte production necessary for early hematopoietic recovery by enhancing G-CSF and IL-6 levels. Based on our findings we propose the use of G-003M as a safe and effective radioprotective strategy to restore GI tract injuries induced by varied kind of occupational, accidental and medicinal exposure to IR. Besides, it may also be explored to rescue normal tissue during anti-cancer radiotherapy.

## Supporting Information

S1 Fig Arrive guideline checklist. The ARRIVE guidelines of animal experimentation was followed for the study.
(PDF)
